# Mid-Term Sequelae of Surviving Patients Hospitalized in Intensive Care Unit for COVID-19 Infection: The REHCOVER Study

**DOI:** 10.3390/jcm12031000

**Published:** 2023-01-28

**Authors:** Marie Berger, Delphine Daubin, Jeremy Charriot, Kada Klouche, Vincent Le Moing, David Morquin, Laurence Halimi, Audrey Jaussent, Patrice Taourel, Maurice Hayot, Jean-Paul Cristol, Nicolas Nagot, Pierre Fesler, Camille Roubille

**Affiliations:** 1 Department of Internal Medicine, Montpellier University Hospital, 34090 Montpellier, France; 2 Critical Care Unit, Montpellier University Hospital, 34090 Montpellier, France; 3 Department of Respiratory Diseases, Montpellier University Hospital, 34090 Montpellier, France; 4 PhyMedExp, INSERM U1046, CNRS UMR 9214, University of Montpellier, 34295 Montpellier, France; 5 Faculty of Medicine, University of Montpellier, 34090 Montpellier, France; 6 Department of Infectious Diseases, Montpellier University Hospital, 34090 Montpellier, France; 7 Clinical Research and Epidemiology Unit, Montpellier University Hospital, 34090 Montpellier, France; 8 Department of Medical Imaging, Montpellier University Hospital, 34090 Montpellier, France; 9 Department of Clinical Physiology, University Hospital of Montpellier, 34090 Montpellier, France; 10 Department of Biochemistry and Hormonology, University Hospital of Montpellier, 34090 Montpellier, France

**Keywords:** SARS-COV-2, COVID-19, mid-term outcomes, sequelae, intensive care

## Abstract

Objectives: The objective of this prospective, single-center study was to explore the mid-term outcomes 6 to 9 months after hospitalization in an Intensive Care Unit (ICU) for severe COVID-19 infection. Methods: Patients systematically underwent biological tests, pulmonary function tests, chest computed tomography (CT) scan, and psychological tests. Results: Among 86 patients, including 71 (82.6%) men, median age of 65.8 years (56.7; 72.4), 57 (71.3%) patients presented post-COVID-19 asthenia, 39 (48.1%) muscle weakness, and 30 (36.6%) arthralgia. Fifty-two (64.2%) patients had a decreased diffusion capacity for carbon monoxide (DLCO) <80% and 16 (19.8%) had DLCO <60%. Chest CT-scans showed ground glass opacities in 35 (40.7%) patients, and reticular changes in 28 patients (33.7%), including fibrosis-like changes in 18 (21.7%) patients. Reticular changes and DLCO <60% were associated with length of stay in ICU, and reticular changes with higher maximal CRP level. The psychological questionnaires found 37.7% suffered from depression, 23.5% from anxiety, 42.4% from insomnia, and 9.4% from post-traumatic stress. Being female was associated with a higher frequency of depression and anxiety, with depression scores being associated with obesity. Conclusions: Many patients hospitalized in ICU for severe COVID-19 infection have mid-term sequelae. Additional studies on the prognostic factors seem necessary.

## 1. Introduction

In December 2019, following the description of Acute Respiratory Distress Syndrome (ARDS) cases in the Wuhan region of China, a new coronavirus was identified: SARS-COV-2 [1], leading to the Coronavirus Disease-19 (COVID-19). During the first waves, approximatively 3% of patients in the general population and 40% of hospitalized patients would develop ARDS within a median of 8 days after the first symptoms [2], requiring hospitalization in an intensive care unit (ICU). The estimated overall mortality rate was around 0.66% (0.39–1.33) [3] and the inpatient mortality was 15%, which is three times higher than that for seasonal flu (5.8%) [4]. A recent systematic review reported a mortality rate of over 30% in ICU [5]. The main risk factors of severity and death of COVID-19 are age [3] and comorbidities. A report from the China Center for Disease Control (CCDC) analyzing 72,314 cases identified a mortality rate of 10.5% for cardiovascular diseases, 7.3% for diabetes, 6.3% for chronic respiratory disease, 6.0% for hypertension, and 5.6% for cancer [6]. The combination of comorbidities increases the risk of death [7].

It seems necessary to investigate possible sequelae of COVID-19, especially for patients who have been hospitalized in ICU, in order to ensure appropriate follow-up as well as anticipate future health needs. The medical community has focused on the mid-term outcomes of COVID-19, organ by organ, suggesting cardiac, pulmonary, and psychiatric sequelae [8]. However, few studies have focused on patients as a whole. Furthermore, as the severity of COVID-19 is associated with the presence of comorbidities, it seems legitimate to screen for them and to review their management after the infection.

The purpose of the present study was to explore the mid-term outcomes of patients hospitalized in ICU for SARS-COV-2 infection by standardized assessment of sequelae and comorbidities within 6 to 9 months post-hospitalization. The primary objective was to describe the incidence of respiratory sequelae and their associated factors. Secondary objectives were to describe COVID-19 worsening or incidence of cardiovascular, metabolic, and psychological comorbidities.

## 2. Patients and Methods

### 2.1. Population

The REHCOVER (Reassessment after Hospitalization for SARS-COV-2 disorder) (NCT 04443257) prospective uncontrolled cohort study included adult patients who were hospitalized in the ICU of the Montpellier University Hospital (France) for COVID-19 from 3rd March 2020 to 21st March 2021. Patients included were over 18 years of age, admitted to the ICU for confirmed SARS-COV-2 infection, proven by positive reverse transcriptase polymerase chain reaction (RT-PCR), and/or typical lesions seen on chest computed tomography (CT) scan associated with clinical signs of SARS-COV-2 infection. Exclusion criteria were patients under legal protection, non-members of the national health insurance, and pregnant or lactating women. The protocol of the REHCOVER study was approved by the ethics committee of Ile de France, Paris VIII, France (No. 20 06 31).

Patients were given information about the study at discharge. They were then contacted to schedule a one-day hospitalization visit performed within 6 to 9 months after the ICU hospitalization. All included patients provided written and signed informed consent.

### 2.2. Data Collection

The collection of events regarding the acute episode of COVID-19 was based on patient medical records, including age, gender, the highest level of respiratory support (invasive ventilation versus non-invasive ventilation including both high-flow nasal oxygen and non-invasive positive pressure ventilation) and duration of respiratory support, length of hospital stay and of ICU stay, and clinical and biological data. Comorbidities were identified and summarized by the Charlson’s index [9]. Patients were asked about the following post-COVID-19 events or symptoms: diabetes imbalance, blood pressure imbalance, acute coronary syndrome, heart failure flare, thromboembolic event, asthenia, arthralgia, muscle weakness, and persistent anosmia. Diabetes imbalance, blood pressure imbalance, and heart failure flare were retained when treatment for these chronic conditions had to be modified after COVID-19 infection. The response was scored “yes” if the patient had experienced this event or symptom after discharge, whether or not it was resolved at the time of assessment. Patients underwent a complete detailed clinical examination and biological tests, including creatininemia, liver tests, ferritin level, NT-proBNP, HbA1C, vitamin D, and SARS-COV-2 serology (IgG and IgM).

#### 2.2.1. Respiratory Assessment

Patients were asked to rate their current dyspnea using the Medical Research Council (MRC) scale [10]. A 6-min walk test was performed with assessment of dyspnea using the Borg scale and oxygen saturation before and after the test [11]. Patients systematically underwent pulmonary function tests (PFT) (spirometry, plethysmography, diffusion capacity for carbon monoxide [DLCO]) and high-resolution non-contrast chest computed tomography (CT) scan, including some CT images with respiratory gating in prone position.

#### 2.2.2. Cardiac Assessment

All patients were assessed with trans-thoracic cardiac ultrasound, electrocardiogram, and NT-proBNP level.

#### 2.2.3. Psychological Assessment

A clinical research associate (CRA) administered questionnaires exploring quality of life and fatigue (Life Orientation Test-Revised (LOT-R) [12], Fatigue Severity Scale (FSS) [13] and EQ-5D-3L) as well as psychological sequelae, using Patient Health Questionnaire-9 (PHQ-9) [14], Generalized Anxiety Disorder-7 (GAD-7) [15], Post-traumatic Stress Disorder Checklist (PCL5) [16], Insomny Severity Index (ISI) [17], Cut down drinking Annoyed by criticism Guilty feelings Eye-opener (CAGE) [18], and Analog Visual Scale (AVS) moral pain (Supplementary Table S1).

In case of clinical and/or paraclinical abnormality, the examiners were able to organize the necessary medical investigations and follow-up.

### 2.3. Statistical Analysis

Demographic and clinical characteristics of patients assessed during and within 6 to 9 months after ICU hospitalization were described in numbers and percentages for categorical variables, and in median with the first (Q1) and third (Q3) quartile of the distribution for quantitative variables because their distributions were tested with the Shapiro–Wilk statistic and were mostly skewed.

Associations with mid-term pulmonary sequelae (DLCO < 60%, presence of reticular changes) and psychological sequelae (PHQ-9 score ≥ 5, GAD-7 score > 5) of COVID-19 infection after ICU hospitalization were quantified with odds ratios (OR) and 95% confidence intervals (CI). To determine which factors were independently associated with each sequela, the factors associated in the univariate analysis at *p* < 0.20 were proposed in a multivariate logistic regression model. A forward selection was applied using the Akaike Information Criterion (AIC).

The significance level was set at *p* < 0.05. Analyses were performed using SAS (version 9.4; SAS Inc., Cary, NC, USA).

## 3. Results

**Figure 1:**
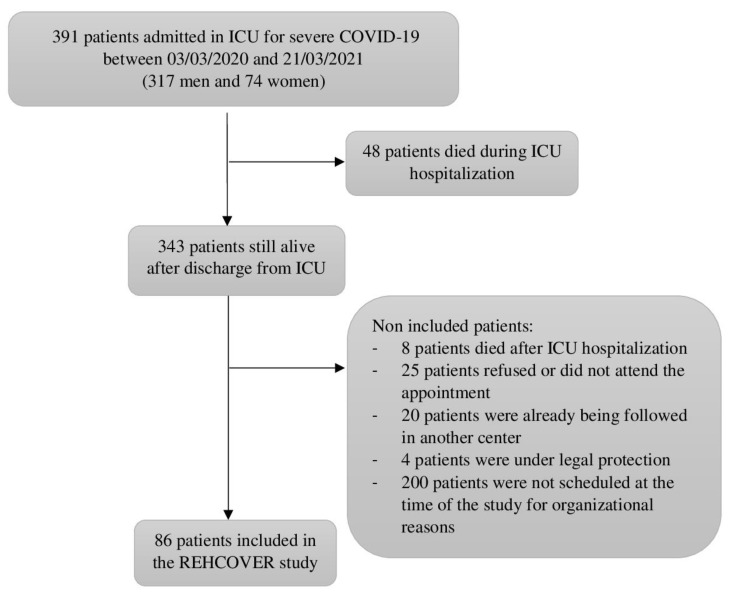
Study design. Abbreviations: ICU: intensive care unit; COVID-19: Coronavirus Disease-19.

Eighty-six patients were included in the study (Figure 1), including 71 men (82.6%) and 15 women (17.4%), with a median age of 65.8 years (56.7; 72.4). Most patients were overweight (*n* = 71, 82.6%), with a median BMI of 29.9 (26.3; 32.4). The median length of hospital stay was 15.5 (11; 24) days, and the median length of stay in ICU was 10.0 (6; 17) days. All included patients had acute respiratory distress syndrome (ARDS) according to the Berlin definition [19]. Fifty-four (62.8%) patients required invasive ventilation, for a median duration of 9 days (5; 14).

Patients’ characteristics at baseline and during hospitalization are summarized in Table 1. Obesity (BMI > 30 kg/m^2^), hypertension, diabetes, and dyslipidemia were the most common pre-existing comorbidities with a frequency of 50%, 46.5%, 34.9%, and 39.5%, respectively. Twelve patients (14.0%) had an underlying, already diagnosed pulmonary disease (including seven patients with chronic obstructive pulmonary disease [COPD], two patients with emphysema, one patient with asthma, one patient with bronchiolitis obliterans with pneumonia organization, and one patient with pre-existing pulmonary fibrosis). Six patients were active smokers, 42 were former smokers, and 36 had never smoked. The median Charlson’s comorbidity index was 1 (0; 2).

The assessment for the present study took place, on average, 7.2 months (±1.9 months) after discharge from the ICU. Fifty-seven (71.3%) patients reported post-COVID-19 asthenia, 39 patients (48.1%) reported muscle weakness, 30 patients (36.6%) reported arthralgia, and 8 (9.8%) patients reported persistent anosmia. Fifteen patients had a diabetic imbalance (which represents 44.8% of diabetic patients), nine had uncontrolled hypertension, eight had a thromboembolic event, five had a heart failure flare, and one had acute coronary syndrome.

### 3.1. Respiratory Assessment

The median distance traveled on the 6-min walk test was 520 m (416; 590) and no significant desaturation was noted. The distance was less than 80% of the predicted value for 17 (21.3%) patients. Most patients did not report significant dyspnea (MRC score 0 for 46 patients (53.5%) and 1 for 24 patients (27.9%)) (Table 2). Sixteen patients had an MRC score ≥2, including 11 men (69%), mainly multimorbid patients (median of number of comorbidities 3 [2–4.5]), with 13 overweight patients and three patients having an underlying, already diagnosed pulmonary disease. Among these 16 patients, six had a DLCO <60% and five had fibrosis.

Seventy (83.3%) patients had normal auscultation. Fourteen (16.3%) patients had abnormal auscultation, mainly dry-base crackles.

Regarding PFT (Table 2), the median lung diffusion capacity for carbon monoxide (DLCO) was 73% (61; 84). Fifty-two (64.2%) patients had DLCO <80% and 16 (19.8%) had DLCO <60%. Among these 16 patients with DLCO < 60%, six patients had an MRC score ≥2, and six had an underlying, already diagnosed pulmonary disease.

**Figure 2:**
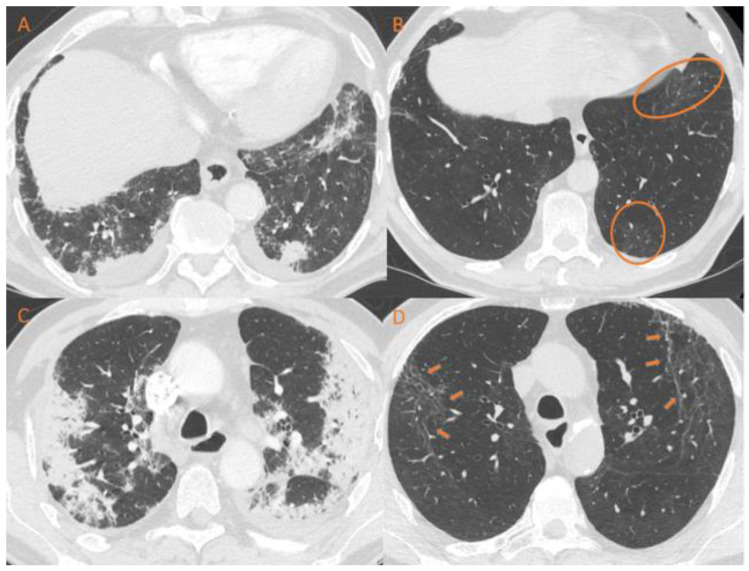
Example of CT-scan evolution in a 76-year-old patient. (**A**,**C**) acute COVID-19 phase. (**B**,**D**) CT-scan performed 7 months later showing persistent ground glass opacities (**B**) and retractile reticulations (**D**).

Chest CT-scan showed consolidation in 22 (25.6%) patients, ground-glass opacities in 35 (40.7%) patients, and reticular changes in 28 patients (33.7%), including fibrosis-like changes in 18 (21.7%) patients. Lesions were predominantly subpleural in 36 (41.9%) patients. Figure 2 shows an example of a CT-scan changes.

In univariate analysis, the risk of decreased DLCO < 60% was associated with a longer length of stay in ICU and a higher maximal C-reactive protein (CRP) level (Table 3). Reticular changes including fibrosis were significantly associated with an older patient’s age, a longer length of stay in ICU, and a higher maximal CRP level (Table 4). Dyspnea as assessed by MRC score was associated with fibrosis and DLCO <60% (*p* < 0.01). In multivariate analysis, both decreased DLCO and reticular changes including fibrosis were significantly associated with a longer length of stay in ICU, independently of covariates. Moreover, reticular changes including fibrosis were significantly associated with a higher maximal CRP level, independently of covariates. Pulmonary sequelae were not associated with sex, obesity, Charlson’s index, and the type of respiratory assistance.

### 3.2. Psychological Assessment

Regarding the psychological questionnaires, 32 patients (37.7%) had at least a mild level of depression on the PHQ-9 score and 14 (16.5%) had moderate or severe depression (Table 2). An anxiety disorder was evidenced in 20 (23.5%) patients and eight (9.4%) patients had a PCL5 score above the threshold suggesting post-traumatic stress disorder. Insomnia was reported in 36 patients (42.4%). Among patients with PHQ-9 and GAD-7 scores above the threshold for depression and anxiety, 65.6% and 78.9% had insomnia, respectively.

The median fatigue score, assessed by FSS, was 2.5 (0.78; 4.67). Most of patients (94.1%) did not report excessive alcohol consumption.

Being female was significantly associated with a higher frequency of psychological sequelae (association with poor PHQ9 and GAD-7, *p*-values < 0.05) in the multivariate analysis (Table 5 and Table 6). The PHQ-9 score was also associated with obesity in multivariate analysis (Table 5). Psychological sequelae were not associated with the severity of the disease (length of stay in ICU, type of respiratory assistance, and maximal CRP level).

## 4. Discussion

In the REHCOVER study, we aimed to explore the mid-term sequelae of survivors of severe COVID-19 infection after ICU hospitalization. Although most of patients did not report dyspnea, 64.2% had a decrease in DLCO <80% and 19.8% experienced a decrease of <60%. Chest CT-scan showed persistent abnormalities such as ground-glass lesions in 40.7% of patients and fibrosis in 21.7%, which is consistent with previous studies. Reticular changes including fibrosis-like changes were associated with the length of stay in ICU, and peak CRP level during the acute episode. DLCO <60% was associated with the length of stay in ICU. The presence of psychological sequelae, systematically assessed, were common, with 37.7% of patients showing symptoms of depression, 23.5% anxiety, and 9.4% post-traumatic stress disorder (PTSD).

The originality of this work is that it concerns only patients hospitalized in ICU and that to a standardized and complete somatic evaluation was added a psychological evaluation, allowing us to evaluate the patients in their globality. Although the prevalence of sequelae is difficult to compare across cohorts because they differ in the time from evaluation to acute COVID-19, the proportion of patients admitted to the ICU, the patients’ maximum level of ventilatory support, and the pharmacotherapy administered in the acute phase, our results are consistent with some of the major studies evaluating post-COVID-19 pulmonary sequelae in ICU inpatients [20,21,22,23,24,25,26,27,28,29,30,31,32,33,34,35,36,37]. When we focus on the studies evaluating patients at 4 months or longer [20,21,22,23,24,25], we find, as in our cohort, a median age between 55 and 65 years, a male predominance, and a tendency to be overweight. In these studies, as in ours, the most common medical history was hypertension followed by diabetes.

As regards respiratory sequelae, the decrease in DLCO is the most frequent abnormality in PFT. This decrease in DLCO is mostly moderate but concerns more than half of patients. In our study, the frequency of respiratory damage (on PFT and CT-scan) contrasts with the moderate respiratory complaint and normal lung auscultation. These results are again consistent with other large studies [21,24]. In our opinion, this reinforces the need for a systematic and standardized evaluation of mid-term respiratory sequelae in patients hospitalized for severe COVID-19 in ICU, because we are currently unable to say whether these pauci-symptomatic lesions will not have an impact on morbidity and mortality in the longer term.

The respiratory manifestations observed in our patients could be secondary to the ICU stay, the association of these manifestations being compatible with a post-intensive care syndrome [38], favored by ventilation [39,40], sedation, and prolonged curarization. Our results showed, in agreement with this, an association between decrease in DLCO and reticular changes with the length of stay in ICU. However, we did not find any association between respiratory sequelae and the type of respiratory assistance, with the possibility of sequelae also in patients under non-invasive ventilation, suggesting a virus-specific effect, at least in part, which is consistent with other studies that have shown the presence of mid-term sequelae in patients with less severe COVID-19 [21,24,28]. Several studies have suggested the existence of virally mediated fibrotic pathways. In the case of COVID-19, binding to the ACE2 receptor may be responsible for a decrease in its anti-fibrotic activity [41]. Additionally, the cytokine storm seen in COVID-19 could be damaging [42], and interestingly, we found a significant association between the peak level of CRP and the mid-term presence of reticular changes and decreased DLCO.

A significant proportion of patients had uncontrolled hypertension after COVID-19. This could be due to a propensity of hypertensive patients to have severe COVID-19 [6] or to discontinuation of therapy during hospitalization. In contrast to the acute phase [2,43], our study did not reveal a high frequency of post-COVID-19 cardiac events or significant cardiac involvement on ultrasound. Several studies have shown excess mortality in patients with cardiac involvement during COVID-19 [44,45]. Consequently, the low proportion of cardiac sequelae in our cohort could be due to patient death before re-evaluation.

A significant proportion of patients developed post-COVID-19 diabetes imbalance. At the time of the reassessment, 17 patients had an HbA1C outside the targets and diabetes was discovered in two patients (HbA1C > 6.5%). This type of complication can be secondary to the corticosteroid therapy administered during acute COVID-19, the interruption of the usual antidiabetic therapy during the acute phase, and to the inflammation induced by the cytokine storm. Some authors have suggested the possibility of an alteration of pancreatic B cells by SARS-COV-2 [46]. A similar phenomenon was observed for SARS-COV-1 infection [47]. These results suggest that careful monitoring of HBA1C should be performed after severe COVID-19.

Our study revealed a high frequency of post-COVID-19 symptoms, with a predominance of asthenia, muscle weakness, and arthralgia. This is consistent with the data in the literature which report frequent and multiple manifestations in post-COVID-19 [21,24,38,48,49].

The level of quality of life after the acute episode was satisfactory with a median of the EQ-5D-3L at 0.89 (0.64; 1.00). Indeed, the mean EQ-5D-3L score in France is estimated to be 0.884 for the 55–64 age group and 0.865 for the 65–74 age group [50]. The quality of life was better than that observed in other studies for COVID-19 [24,29,51] and for MERS-COV and SARS-COV-1 [52]. This may reflect the satisfactory level of rehabilitation [53], the relatively asymptomatic nature of the respiratory involvement, or selection bias. Further studies are needed to explore this point.

In addition, we found some mid-term psychological sequelae of COVID-19 infection, including depression (37.6%), anxiety (23.5%), and PTSD (9.4%), mostly mild to moderate. The frequency of psychiatric disorders was higher than those observed, retrospectively, on computerized records in another study, with 15.4% mood disorders, 19.2% anxiety disorders, and 7.5% insomnia [54]. However, it should be kept in mind that the psychological sequelae in particular may have been assessed differently than in our study, either with other questionnaires or with ICD codes, which may in itself explain the differences. In any case, this reinforces the need for systematic screening for post-COVID-19 psychiatric disorders.

The high prevalence of post-COVID-19 psychiatric disorders may also be secondary to hospitalization in ICU. However, the higher risk of depression and anxiety reported in patients hospitalized for COVID-19 compared with influenza or other respiratory tract infections suggest the specific influence of SARS-COV-2 on psychiatric disorders [54,55]. A significant proportion of patients (9.41%) showed a post-traumatic stress disorder. A higher rate of 25–30% [56,57] has been reported in the literature. However, this assessment was performed much earlier. Nevertheless, it seems interesting to realize that post-traumatic stress can persist at a distance as in our study.

Many patients suffered from insomnia after the acute episode. Post-infection sleep disorders have been frequently reported in the literature [21,24]. It remains unclear whether this insomnia may be a neurological impairment of COVID-19, or whether it is secondary to psychiatric disorders such as depression or anxiety, as most patients with anxiety or depression were also found to have insomnia.

## 5. Limitations

Our study has several limitations. First, this is an uncontrolled cohort study, without a non-COVID-19 control group. Not all patients were assessed at exactly the same time after hospitalization, and this may represent a bias because abnormalities may tend to improve over time. We were not able to exhaustively assess all patients hospitalized in ICU; however, no selection of patients was made, and we believe that our study population is representative of all patients who had been admitted in ICU for severe COVID-19 infection. The chest CT-scan was not blinded in the study and did not benefit from a second reading. However, the agreement with the prevalence of abnormalities in other studies [21,23,24] suggests reliable results. In addition, patients did not benefit from a psychiatrist evaluation, and we had no data on their psychological state before COVID-19 infection (previously diagnosed depressive state, treated or not, or latent undiagnosed). However, the psychological questionnaires were chosen for their ease of completion and their validity, thus allowing the screening of several pathologies during the same consultation. Finally, if the tests revealed any disorder, patients could benefit from a consultation with a psychologist or a psychiatrist (depending on the severity of the score).

To summarize, in this prospective study of 86 patients hospitalized in ICU for severe COVID-19 infection, we found a large proportion of patients with mid-term sequelae. Respiratory function tests were pathological in more than half of the patients with a decrease in DLCO. Chest CT-scan showed ground-glass opacities in 40.7% of patients and fibrosis in 21.7%. These abnormalities contrasted with a moderate respiratory complaint and a normal clinical examination, arguing for a systematic evaluation in these patients. Non-respiratory symptomatology was varied, with a significant prevalence of asthenia, muscle weakness and arthralgia, and psychological disorders.

It seems that special attention should be paid to patients with a long stay in ICU (reflected by an association between DLCO, fibrosis, and length of hospitalization). Further studies are needed to find prognostic factors for sequelae, such as the influence of acute-phase treatment. The sequelae of COVID-19 are probably multifactorial in origin, combining personal susceptibility, critical care factors, and a specific viral effect. Beyond a simple description of post-COVID-19 sequelae, this study invites us to adopt a proactive attitude in the management of these patients through specialized follow-up.

## Figures and Tables

**Table 1:** Baseline and hospitalization characteristics of the patients.

Demographic Characteristics and Comorbidities	*n*	No. (%)
Age (years) ^a^	86	65.8 (56.7; 72.4)
≥50 yrs		76 (88.4)
<50 yrs		10 (11.6)
Sex	86	
Female		15 (17.4)
Male		71 (82.6)
Comorbidities	86	
Hypertension		40 (46.5)
Diabetes		30 (34.9)
Dyslipidemia		34 (39.5)
Ischemic heart disease		10 (11.6)
Heart failure		7 (8.1)
Vascular disease		4 (4.7)
Systemic disease		3 (3.5)
Stroke		2 (2.3)
Chronic pulmonary disease		12 (14.0)
Gastric ulcer		8 (9.3)
Liver disease		9 (10.5)
Chronic kidney failure		10 (11.6)
Malignancy		15 (17.4)
Sleep apnea		20 (23.3)
Depression		13 (15.1)
Obesity (BMI > 30 Kg/m^2^)		43 (50.0)
Charlson’s index ^a^		1 (0; 2)
Smoking status	84	
Active smokers		6 (7.1)
Never smokers		36 (42.9)
Former smokers		42 (50.0)
Acute phase of COVID-19		
Length of hospital stay (days) ^a^	86	15.5 (11; 24)
Length of ICU stay (days) ^a^	86	10 (6; 17)
Specific treatments	86	
Corticosteroids		67 (77.9)
Lopinavir/ritonavir		16 (18.6)
Type of respiratory assitance	86	
Non-invasive ventilation		32 (37.2)
Invasive/mechanical ventilation		54 (62.8)
Length of invasive ventilation (days) ^a^	54	9 (5;14)
Length of invasive ventilation > 7 days (*n*, %)		33 (61.1)
Length of invasive ventilation > median (9 days)(*n*, %)		24 (44.5)
SOFA score ^a^	71	5 (2;7)
Loss of weight (Kg) in the last 6 months ^a^	73	5 (0;10)
Clotting disorder	86	17 (19.8)
Pulmonary embolism	86	12 (13.9)
Anosmia	75	33 (44.0)
Lymphopenia	86	74 (86.0)
Abnormal liver function test	86	77 (89.5)
Minimal GFR (mL/Min) ^a^	86	63.50 (47.00; 93.00)
Maximal creatininemia (µmol/L) ^a^	86	100.50 (75.00; 135.00)
Maximal CRP level (mg/L) ^a^	86	234.50 (163.00; 292.00)

^a^: Quantitative variables are expressed as median (quartile 1–quartile 3). Abbreviations: CRP: C-Reactive Protein; GFR: Glomerular Filtration Rate.

**Table 2:** Results of the REHCOVER visit.

Clinical Characteristics	*n*	No. (%)
Weight (Kg) ^a^	86	85 (76; 97)
Height (m) ^a^	86	1.73 (1.67; 1.78)
BMI (Kg/m^2^) ^a^	86	29.9 (26.3; 32.4)
Systolic blood pressure (mmHg)	77	130 (116; 138)
Diastolic blood pressure (mmHg)	77	75 (67; 81)
Pulmonary Auscultation		
Crackles	84	13 (15.5)
Bronchial rales	84	1 (1.2)
6-min walk test		
Distance covered ^a^	80	520 (416;590)
Distance < 80%	80	17 (21.3)
Borg scale after	78	3 (1; 4)
Dyspnea, MRC score	86	
MRC 0		46 (53.5)
MRC 1		24 (27.9)
MRC 2		13 (15.1)
MRC 3		3 (3.5)
MRC 4		0 (0)
Muscular weakness	81	39 (48.1)
Pulmonary function tests		
FEV (mL) ^a^	86	3035 (2420; 3530)
FEV (% of predicted value) ^a^	86	104.50 (92; 116)
FVC (% of predicted value) ^a^	86	100.50 (86; 113)
FEV/FVC < 70	86	6 (7)
DLCO (%) ^a^	81	73 (61; 84)
DLCO < 80%		52 (64.2)
DLCO < 60%		16 (19.8)
Chest CT-scan		
Consolidation	86	22 (25.6)
Persistent ground glass opacities	86	
All		35 (40.7)
Large area		13 (15.1)
Nodular		22 (25.6)
Reticular changes (including fibrotic lesions)	83	28 (33.7)
Reticulations	82	27 (32.9)
Lung Fibrotic lesions	83	18 (21.7)
Subpleural location	86	36 (41.9)
Echocardiography assessment		
Preserved LVEF	80	71 (88.8)
LVEF (%) ^a^	70	60 (55;60)
Wall motion abnormality	80	8 (10)
Pericardial effusion	77	2 (2.6)
Biological		
GFR	86	
<60 mL/min/1.73 m^2^		17 (19.8)
[60; 90] mL/min/1.73 m^2^		41 (47.7)
>90 mL/min/1.73 m^2^		28 (32.6)
HbA1C (%) ^a^	83	6.1 (5.7; 6.8)
Vitamin-D (nmol/mL) ^a^	82	69.5 (44; 97)
Liver test (UI/mL)		
PAL ≥ 105 UI/L	86	8 (9.3)
GGT ≥ 40 UI/L	86	36 (41.9)
TLAT ≥ 33 UI/L	85	11 (12.9)
TSAT ≥ 32 UI/L	85	8 (9.4)
Ferritin (µg/L) ^a^	85	176 (84; 353)
NT-Pro BNP ≥ 300 ng/L	81	14 (17.3)
IgG anti-N anti SARS-COV-2 positive	76	70 (92.1)
Psychological assessment		
PHQ-9	85	
No depression		53 (62.4)
Mild severity of depression		18 (21.2)
Moderate severity of depression		8 (9.4)
Severe severity of depression		6 (7.1)
GAD 7	85	
No anxiety		65 (76.5)
Mild level of anxiety		12 (14.1)
Moderate level of anxiety		7 (8.2)
Severe level of anxiety		1 (1.2)
PCLS	85	
<44: No PTSD		77 (90.6)
≥44: possible PTSD		8 (9.4)
ISI	85	
Absence		49 (57.6)
Mild		16 (18.8)
Moderate		18 (21.2)
Severe		2 (2.4)
FSS ^a^	86	2.50 (0.78; 4.67)
CAGE	85	
Score: 0–1		80 (94.1)
Score 2–4		5 (5.9)
LOT-R (0–24) ^a^	85	17 (13; 22)
EVA psychological pain (/10) ^a^	86	
Current		0.5 (0; 5)
During acute COVID-19		5 (0; 9)
Before COVID-19		0 (0; 1)
EQ-5D-3L ^a^	85	0.89 (0.64; 1.00)

^a^: Quantitative variables are expressed as median (quartile 1–quartile 3). Abbreviations: FEV: Forced Expiratory Flow; AVS: Analog Visual Scale; CAGE: Cut down drinking Annoyed by criticism Guilty feelings Eye-opener; EVA: Echelle Visuelle Analogique; FSS: Fatigue Severity Scale (FSS); GAD-7: Generalized Anxiety Disorder-7; GFR: Glomerular Filtration Rate; ICU: Intensive Care Unit; ISI: Insomnia Severity Index; LOT-R: Life Orientation Test—Revised; PCL5: Posttraumatic Stress Disorder Checklist., PHQ9: Patient Health Questionnaire-9. PTSD: post-traumatic stress disorder.

**Table 3:** Factors associated with decreased DLCO < 60%.

*n* = 81	DLCO < 60% (Y/N)		Unit (for OR)	Univariate Analysis		Multivariate Analysis *	
Variable	NO	YES		Crude Odds Ratio [CI 95%]	*p*-Value	Adjusted Odds Ratio [CI 95%]	*p*-Value
	(*n* = 65)	(*n* = 16)					
Sex (*n*(%))					0.1884		-
Male	51 (78.46)	15 (93.75)		1			-
Female	14 (21.54)	1 (6.25)		0.243 [0.029; 2.001]			
Age					0.1755		-
Mean (SD)	62.95 (11.22)	67.37 (12.67)	10	1.469 [0.842; 2.562]		-	
Median (Q1; Q3)	65.08 (54.41; 71.55)	71.55 (59.73; 76.86)					
Obesity (BMI > 30) (Y/N) (*n* (%))					0.6946		-
No	32 (49.23)	7 (43.75)		1			
Yes	33 (50.77)	9 (56.25)		1.247 [0.415; 3.749]		-	
Charlson’s index (*n* (%))					0.8744		-
0	23 (35.38)	6 (37.50)		1			
1 or more	42 (64.62)	10 (62.50)		0.913 [0.294; 2.833]		-	
Length of ICU stay (days)					0.0008		0.0008
Mean (SD)	13.05 (13.95)	32.06 (19.45)	1	1.062 [1.025; 1.100]		1.062 [1.025; 1.100]	
Median (Q1; Q3)	10.00 (6.00; 14.00)	38.00 (11.00; 49.50)					
SOFA score							
Mean (SD)	4.39 (2.72	6.62 (2.29)	1	1.358 [1.064; 1.733]	0.0141		
Median (Q1; Q3)	3.50 (2.00; 6.00)	8.00 (5.00; 8.00)					
Corticotherapy (*n* (%))					0.2605		-
No	17 (26.15)	2 (12.50)		1			
Yes	48 (73.85)	14 (87.50)		2.479 [0.510; 12.054]		-	
Type of respiratory assistance (*n* (%))					0.1484		-
Non-invasive ventilation	25 (38.46)	3 (18.75)		1			
Invasive ventilation	40 (61.54)	13 (81.25)		2.708 [0.701; 10.459]		-	
Maximal CRP level					0.0415		-
Mean (SD)	223.60 (94.42)	281.86 (108.64)	50	1.354 [1.012; 1.812]		-	
Median (Q1; Q3)	215.50 (158; 285)	273.75 (219.5; 337)					

Abbreviations: BMI: Body Mass Index; CRP: C-Reactive Protein; DLCO: Diffusing Capacity of Carbone monoxide; ICU: Intensive Care Unit; OR: odds ratio; SD: Standard Deviation; CI: Confidence interval * Multivariate analysis after selection of the best model via minimal AIC, AIC = 70.457, R^2^ = 0.2528, Test Hosmer & Lemeshow *p* = 0.8883, AUC = 0.7793 [0.6360; 0.9227].

**Table 4:** Factors associated with reticular changes including fibrosis.

*n* = 83	Reticular Changes (Y/N)		Unit (for OR)	Univariate Analysis		Multivariate Analysis *	
Variable	NO	YES		Crude Odds Ratio [CI 95%]	*p*-Value	Adjusted Odds Ratio [CI 95%]	*p*-Value
	(*n* = 55)	(*n* = 28)					
Sex (*n* (%))					0.2231		-
Male	43 (78.18)	25 (89.29)		1		-	
Female	12 (21.82)	3 (10.71)		0.430 [0.111; 1.672]			
Age					0.0196		0.0976
Mean (SD)	61.77 (11.27)	68.21 (10.78)	10	1.816 [1.100; 2.996]		1.649 [0.912; 2.979]	
Median (Q1; Q3)	63.13 (53.84; 70.89)	70.51 (65.96; 75.94)					
Obesity (BMI > 30) (Y/N) (*n* (%))					0.8185		-
No	27 (49.09)	13 (46.43)		1			
Yes	28 (50.91)	15 (53.57)		1.113 [0.447; 2.769]		-	
Charlson’s index (*n*(%))					0.7031		-
0	20 (36.36)	9 (32.14)		1		-	
1 or more	35 (63.64)	19 (67.86)		1.206 [0.460; 3.166]		-	
Length of ICU stay (days)					0.0008		0.0104
Mean (SD)	11.45 (10.31)	26.71 (21.81)	1	1.068 [1.028; 1.110]		1.049 [1.011; 1.089]	
Median (Q1; Q3)	9.00 (6.00; 13.00)	16.50 (11.00; 43.00)					
SOFA score	*n* = 46	*n* = 23			0.3391		
Mean (SD)	4.59 (2.72)	5.26 (2.86)	1	1.093 [0.911; 1.312]			
Median (Q1; Q3)	4.50 (2.00; 7.00)	6.00 (2.00; 8.00)					
Corticotherapy (*n* (%))					0.4383		-
No	14 (25.45)	5 (17.86)		1			
Yes	41 (74.55)	23 (82.14)		1.571 [0.501; 4.919]		-	
Type of respiratory assistance (*n* (%))					0.0704		
Non-invasive ventilation	23 (41.82)	6 (21.43)		1			
Invasive ventilation	32 (58.18)	22 (78.57)		2.635 [0.922; 7.529]		-	
Maximal CRP level					0.0015		0.0122
Mean (SD)	207.90 (88.54)	285.98 (99.02)	50	1.579 [1.190; 2.096]		1.508 [1.094; 2.079]	
Median (Q1; Q3)	211.00 (137; 271)	284.35 (212.5; 357.3)					

Abbreviations: BMI: Body Mass Index; CRP: C-Reactive Protein; ICU: Intensive Care Unit; SD: Standard Deviation; CI: Confidence interval. * Multivariate analysis after selection of the best model via minimal AIC, AIC = 87.238, R^2^ = 0.3834, Hosmer & Lemeshow Test *p* = 0.5106, AUC = 0.8331 [0.7398; 0.9265].

**Table 5:** Factors associated with depression.

*n* = 85	PHQ-9		Unit (for OR)	Univariate Analysis		Multivariate Analysis	
Variable	<5	>=5		Crude Odds Ratio [CI 95%]	*p*-Value	Adjusted Odds Ratio [CI 95%]	*p*-Value
	(*n* = 53)	(*n* = 32)					
Sex (*n* (%))					0.0037		0.0011
Male	49 (92.45)	21 (65.63)		1		1	
Female	4 (7.55)	11 (34.38)		6.416 [1.832; 22.470]		10.427 [2.559; 42.487]	
Patient’s age					0.4670		-
Mean (SD)	63.24 (11.67)	65.12 (11.45)	10	1.157 [0.781; 1.714]		-	
Median (Q1; Q3)	65.66 (57.97; 72.22)	68.19 (53.14; 73.08)					
Obesity (BMI > 30) (Y/N) (*n* (%))					0.0632		0.0144
No	31 (58.49)	12 (37.50)		1		1	
Yes	22 (41.51)	20 (62.50)		2.348 [0.954; 5.777]		3.799 [1.304; 11.066]	
Charlson’s index (*n* (%))					0.1717		0.1516
0	21 (39.62)	8 (25.00)		1		1	
1	32 (60.38)	24 (75.00)		1.968 [0.745; 5.198]		2.218 [0.746; 6.593]	
Length of ICU stay (days)					0.8121		-
Mean (SD)	15.49 (17.36)	16.34 (14.03)	1	1.003 [0.976; 1.031]		-	
Median (Q1; Q3)	9.00 (6.00; 15.00)	12.00 (8.50; 17.00)					
Corticotherapy (*n* (%))					0.1308		-
No	9 (16.98)	10 (31.25)		1			
Yes	44 (83.02)	22 (68.75)		0.450 [0.160; 1.268]		-	
Type of respiratory assistance (*n* (%))					0.1622		-
Non-invasive ventilation	23 (43.40)	9 (28.13)		1			
Invasive ventilation	30 (56.60)	23 (71.88)		1.959 [0.763; 5.029]		-	
Maximal CRP level					0.9681		-
Mean (SD)	233.59 (103.52)	232.72 (92.20)	50	0.995 [0.796; 1.245]			
Median (Q1; Q3)	225 (150.00; 299.00)	240 (178.25; 291.90)				-	

Abreviations: BMI: Body Mass Index; CRP: C-Reactive Protein; ICU: Intensive Care Unit; PHQ-9: Patient Health Questionnaire; SD: Standard Deviation; CI: Confidence interval.

**Table 6:** Factors associated with anxiety.

*n* = 85	GAD-7		Unit (for OR)	Univariate Analysis		Multivariate Analysis	
Variable	<5	>=5		Crude Odds Ratio [CI 95%]	*p*-Value	Adjusted Odds Ratio [CI 95%]	*p*-Value
	(*n* = 65)	(*n* = 20)					
Sex (*n* (%))					0.0152		0.0147
Male	58 (89.23)	13 (65.00)		1		1	
Female	7 (10.77)	7 (35.00)		4.462 [1.333; 14.932]		4.854 [1.364; 17.272]	
Patient’s age					0.0407		0.0963
Mean (SD)	65.17 (11.15)	58.98 (11.66)	10	0.638 [0.415; 0.981]		0.678 [0.429; 1.072]	
Median (Q1; Q3)	66.57 (61.42; 73.09)	57.98 (51.17; 69.91)					
Obesity (BMI > 30) (Y/N) (*n* (%))					0.3381		-
No	34 (52.31)	8 (40.00)		1			
Yes	31 (47.69)	12 (60.00)		1.645 [0.594; 4.555]		-	
Charlson’s index (*n* (%))					0.6150		-
0	22 (33.85)	8 (40.00)		1			
1 or more	43 (66.15)	12 (60.00)		0.767 [0.274; 2.153]		-	
Length of ICU stay (days)					0.0847		0.1562
Mean (SD)	18.11 (18.28)	10.20 (6.83)	1	0.950 [0.896; 1.007]		0.952 [0.889; 1.019]	
Median (Q1; Q3)	10.00 (7.00; 18.00)	10.00 (6.00; 12.50)					
Specific treatment: corticotherapy (Y/N) (*n* (%))					0.0902		-
No	11 (16.92)	7 (35.00)		1			
Yes	54 (83.08)	13 (65.00)		0.378 [0.123; 1.165]		-	
Type of respiratory assistance (cl) (*n* (%))					0.8039		-
Non-invasive ventilation	24 (36.92)	8 (40.00)		1			
Invasive ventilation	41 (63.08)	12 (60.00)		0.878 [0.315; 2.451]		-	
Maximal CRP level					0.1065		-
Mean (SD)	242.29 (100.36)	201.27 (87.39)	50	0.796 [0.603; 1.050]			
Median (Q1; Q3)	246 (168.00; 298.00)	195.80 (143.50; 273.45)				-	

Abreviations: BMI: Body Mass Index; CRP: C-Reactive Protein; GAD-7: Generalized Anxiety Disorder; ICU: Intensive Care Unit; SD: Standard Deviation CI: Confidence interval.

## Data Availability

Data could be available on reasonable request.

## Supplementary Materials

The following supporting information can be downloaded at: https://www.mdpi.com/article/10.3390/jcm12031000/s1, Table S1: Psychological questionnaires.
