# Supplementary material for: Mid-Term Sequelae of Surviving Patients Hospitalized in Intensive Care Unit for COVID-19 Infection: The REHCOVER Study
Source: J Clin Med. 2023 Jan 28;12(3):1000. doi: 10.3390/jcm12031000 (PMC9917891; doi:10.3390/jcm12031000)
Supplement: Supplementary file 1 [file jcm-12-01000-s001.zip › jcm-2147372-supplementary.pdf]

Supplementary Table S1. Psychological questionnaires

Questionnaire<br>Diagnostic ability<br>(Original article for France)	Number of questions and coding	Score calculation	Interpretation
**GAD7**<br>Anxiety<br>[15]	**7**<br>Each question is coded from 0 (never) to 3 (nearly every day)	Sum of the 7 questions	0-4 : Low to no risk<br>5-9 : Mild<br>10-14 : Moderate<br>15-21 : Severe
**PHQ9**<br>Depression<br>[14]	**9**<br>Each question is coded from 0 (never) to 3 (nearly every day)	Sum of the 9 answers	0-4 : None to Minimal<br>5-9 Mild<br>10-14 : Moderate<br>15-19 : Moderately Severe<br>20-27 : Severe
**PCL-5**<br>Post-Traumatic Stress<br>[16]	**17**<br>Each question is coded from 0 (not at all) to 3 (extremely)	Sum of the 17 answers	0-44 : No post-traumatic stress<br>44-85 : Post-traumatic stress
**ISI**<br>Insomnia<br>[17]	**7**<br>Each question is coded from 0 to 4.	Sum of the 7 answers	0-7 : No insomnia<br>8-14 : Subthreshold insomnia<br>15-21 : Moderate clinical insomnia<br>22-28: Severe clinical insomnia
**FSS**<br>Fatigue<br>[13]	**9**<br>Each question is coded from 0 (strongly disagree) to 7 (strongly agree)	Average of the 9 answers	0-3 : Mild or moderate fatigue<br>4-7 : Severe fatigue
**CAGE**<br>Alcohol addiction<br>[18]	**4**<br>Each question is coded from 1= yes and 0= no	Sum of the 4 answers	0 : No addiction<br>1 : At risk<br>2 -3 : Excessive consumption<br>4 : Alcohol addiction
**AVS**<br>Moral pain			Scale from 0 to 10.<br>No cut-off.<br>The higher the score, the more intense the moral pain.
**LOT-R**<br>Life orientation<br>[12]	**10**<br>Each question is coded from 0 (strongly disagree) to 4 (strongly agree)	Inversion of the 3rd, 7th and 9thanswers.<br>Sum of the 1st, 3rd, 4th, 7th, 9th and 10th answers.	Scale from 0 to 24.<br>No cut-off.<br>The higher the score, the more optimistic is the patient.
**EQ5D-3L**<br>Quality of life	**5**<br>Each dimension has 3 levels of severity.	By combining the 5 dimensions, a number is obtained that represents the health status of the patient.	The health status number must then be converted to an index value (0 = death and 1 = full health).
